# A Quantum Chemical and Statistical Study of Phenolic Schiff Bases with Antioxidant Activity against DPPH Free Radical

**DOI:** 10.3390/antiox3020309

**Published:** 2014-04-21

**Authors:** El Hassane Anouar

**Affiliations:** Atta-ur-Rahman Institute for Natural Products Discovery, Level 9, FF3, Universiti Teknologi MARA, Puncak Alam Campus, Bandar Puncak Alam, Selangor Darul Ehsan 42300, Malaysia; E-Mail: anouarelhassane@yahoo.fr; Tel.: +60-332-584-771; Fax: +60-332-584-770

**Keywords:** Schiff bases, antioxidant activity, DFT, MLR, PCA, HCA, QSAR

## Abstract

Phenolic Schiff bases are known as powerful antioxidants. To select the electronic, 2D and 3D descriptors responsible for the free radical scavenging ability of a series of 30 phenolic Schiff bases, a set of molecular descriptors were calculated by using B3P86 (Becke’s three parameter hybrid functional with Perdew 86 correlation functional) combined with 6-31 + G(d,p) basis set (*i.e.*, at the B3P86/6-31 + G(d,p) level of theory). The chemometric methods, simple and multiple linear regressions (SLR and MLR), principal component analysis (PCA) and hierarchical cluster analysis (HCA) were employed to reduce the dimensionality and to investigate the relationship between the calculated descriptors and the antioxidant activity. The results showed that the antioxidant activity mainly depends on the first and second bond dissociation enthalpies of phenolic hydroxyl groups, the dipole moment and the hydrophobicity descriptors. The antioxidant activity is inversely proportional to the main descriptors. The selected descriptors discriminate the Schiff bases into active and inactive antioxidants.

## 1. Introduction

Phenolic Schiff bases have been widely studied due to their various applications in different fields, such as inorganic chemistry, analytical chemistry and biochemistry [1,2]. They are reported as effective corrosion inhibitors on mild steel [3]. They are known for antibacterial [4,5,6,7], anticancer [2], antifungal [8,9] and antileishmanial activities [10]. Due to the presence of phenolic groups, phenolic Schiff bases are reported as powerful antioxidants and good free radical scavengers [11,12]. In general, the free radical scavenging capacity of polyphenols is mainly attributed to the hydrogen atom transfer of the OH, NH and SH groups (attached to aromatic rings) to the free radicals [13,14,15,16]. Hydrogen atom transfer, proton-coupled electron transfer (PC-ET), electron transfer-proton transfer (ET-PT), sequential proton-loss-electron-transfer (SPLET) and adduct formation are the most common mechanisms involved in free radical scavenging. Recently, we showed that the current series of Schiff bases scavenges free radicals through a PC-ET mechanism [13].

Many QSAR studies have attempted to find the relationship between the structural features and the antioxidant activity of polyphenolic compounds [17,18]. The results indicated that some structural features are the most significant in enhancing the antioxidant activity, such as: (i) the presence of a catechol moiety; (ii) the catechol group conjugated to a double bond; and (iii) the number of free hydroxyl groups, as in flavonoids and phenolic acids [17], oligomers of guaiacol [13] and Schiff bases [19]. In addition to the structural features, electronic properties may have an important role in discriminating between more, less and inactive polyphenols. Weber *et al.* found that the polarizability (α) charge at carbons C3, C5 and C3’ as being responsible for the antioxidant activity of flavonoids compounds [20]. It is worth mentioning that the antioxidant activity depends on their reactions with free radicals. Iuga *et al.* studied the antioxidant activity of *trans*-resveratrol toward hydroxyl (^•^OH) and hydroperoxyl (^•^OOH) radicals in aqueous simulated media, using density functional theory (DFT) and transition state theory (TST) methods, and they concluded that the reactivity of *trans*-resveratrol with (i) ^•^OH radical (in water at physiological pH) is mainly governed by a sequential electron proton transfer (SE-ET) mechanism; and with ^•^OOH, it occurs only by phenolic hydrogen abstraction [21]. In another study, Leopoldini *et al.* found that the addition of ^•^OH radical to caffeic acid is slightly favored with respect to the hydrogen atom transfer, while the single electron transfer is unlikely, since thermodynamically, it is unfavorable [22]. Mayer *et al.* and Oksana *et al.* reported that the reactivity of phenols toward free radicals, such as CH_3_OO^•^ and PhO^•^, involved a PC-ET mechanism [23,24].

In this work, density functional theory (DFT) calculations have been carried out at the B3P86/6-31+G(d,p) level of theory to explore and calculate more representative descriptors able to express the electronic, 2D and 3D properties that can be related to the free radical scavenging ability of a series of 30 Schiff bases (Table 1). Furthermore, the pattern recognition methods, such as simple and multiple linear regressions (SLR and MLR), principal component analysis (PCA) and hierarchical cluster analysis (HCA), have been employed with the aim of selecting the variables responsible for the free radical scavenging activity and to describe properly the relationship between the calculated descriptors and the antioxidant activity of these Schiff bases.

## 2. Material and Methods

### 2.1. Synthesis and DPPH Free Radical Scavenging Capacity

The series of phenolic Schiff base were synthesized by Taha *et al.* [10]. The antioxidant capacity was reported in our previous study as the capacity of the 30 Schiff bases to scavenge the 1,1-diphenyl-2-picrylhydrazil (DPPH) free radical [19].

**Table 1:** Molecular structures of the synthesized Schiff bases and their antioxidant activities.

Subclass	No.	Hydroxyl Positions	Other Groups	IC_50 _(μg/mL)
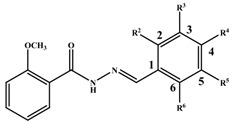	1	2	-	0.90 ± 0.045
	2	3	-	1.1 ± 0.05
	3	4	-	0.65 ± 0.045
	4	2, 3	-	0.22 ± 0.045
	5	2, 4	-	0.34 ± 0.045
	6	2, 5	-	0.92 ± 0.045
	7	3, 4	-	0.20 ± 0.045
	8	3, 5	-	0.91 ± 0.0045
	9	2, 4, 6	-	0.35 ± 0.045
	10	3, 4, 5	-	0.30 ± 0.045
	11	-	3-OCH_3_	>>2
	12	-	4-OCH_3_	>>2
	13	-	3,4-diOCH_3_	>>2
	14	-	3,5-diOCH_3_	>>2
	15	2	4- OCH3	0.50 ± 0.071
	16	2	5-OCH3	1.01 ± 0.045
	17	3	4- OCH3	0.8 ± 0.002
	18	4	3-Br	0.22 ± 0.0045
	19	-	3-Br, 4-Cl	>>2
	20	-	4-F	>>2
	21	-	4-Cl	>>2
	22	3	2-I, 4-OCH_3_	1.63 ± 0.21
	23	-	4-COOCH_3_	>>2
	24	-	4-NO2	>>2
	25	-	-	>>2
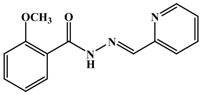	26	-	-	>>2
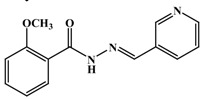	27	-	-	>>2
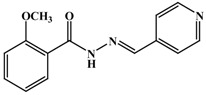	28	-	-	>>2
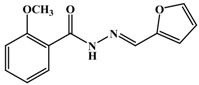	29	-	-	>>2
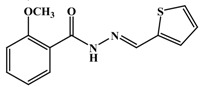	30	-	-	>>2

### 2.2. Theoretical Details

Geometry optimization and frequency calculations of neutral, radical and ionic forms of the minima ground states of Schiff bases have been carried out using the DFT method. The frequency analyses were carried out at the same level of theory. The absence of imaginary frequencies confirmed that the structures are true minima on the potential energy surface. To study the structure-antioxidant activity relationships of polyphenols, different methods (e.g., semiempirical, DFT) can be used. Sakar *et al.* and Mendes *et al.* used B3LYP (Becke’s three parameter hybrid functional with the LYP correlation functional) to study the structure antioxidant activity of flavonoid derivatives [25,26]. In a previous study, we used the hybrid functional B3P86 to study the free radical scavenging properties of guaiacol oligomers [13]. Recently, we used B3P86 and MPWB1K (Modified Perdew and Wang exchange functional (MPW) and Becke’s 1995 correlation functional (B95)) hybrid functionals to study the antioxidant properties and mechanism of actions of the current series of phenolic Schiff bases and hispidin oligomers in DPPH and ^•^OOCH_3_ free radical scavenging [19,27]. In continuity with our previous studies, here we used the hybrid functional B3P86 to calculate the electronic and structural descriptors for the phenolic Schiff bases. Increasing the number of polarization and diffuse functions has no significant effect on the electronic descriptors, such as bond dissociation enthalpies (BDEs) and ionization potentials (IPs) [13]; therefore, the calculations were performed with the double-ζ Pople-type basis set 6-31 + G(d,p). The chemical descriptors that were chosen to be correlated with the antioxidant activity are: the bond dissociation enthalpies related to the first and second hydrogen atom transfer, named BDE and BDEd; the ionization potential for the ArOH and ArO phenoxyl radical, named IP and IPd; electron affinity (EA); electronegativity (χ); hardness (η); softness (S); electrophilicity index (ɷ); molecular polarizability (α); dipole moment (μ); the steric descriptors’ surface area of the molecule (A) and volume (V); molecular weights (M); hydrophobicity (logP), where P stands for the octanol-water partition coefficient; the number of OH groups (nOH); the spin density of the active OH groups (SD); and the free enthalpy of the reaction of the reactivity of phenolic Schiff bases with the DPPH radical (ΔG). The logP values were carried out using the Hyperchem Molecular package [28] by means of the atomic parameters derived by Ghose, Pritchett and Crippen and later extended by Ghose and co-workers [29,30]. The electronic descriptors can be calculated by using three different approaches [31,32,33,34,35,36]. The first approach is based on the use of the classical finite difference approximation, in which the change of one electron is usually involved ΔN = ±1 [31]. In this approach, EA = E_0_ − E_−1_ and IP = E_+1_ − E_0_, where E_0_, E_−1_ and E_+1_ are the electronic energies of a neutral molecule, when adding and removing an electron to the neutral molecule. The second approach is based on Koopman’s theorem, in which IP = −E_HOMO_ and EA = −E_LUMO_. The third approach, named the internally resolved hardness tensor (IRHT), is also based on orbital energies [32,33], and it deals with fractional occupation numbers based on Janak’s extension of DFT [34]. De Luca *et al.* applied the three approaches to study solvent effects on the hardness values of a series of neutral and charged molecules, and they found that the three methods gave similar results in the presence of solvent [35]. Here, the electronic descriptors were calculated in solvent using the Koopman theorem, where IP = −E_HOMO_ and EA = −E_LUMO_. The solvent effects were taken into account implicitly by using the polarizable continuum model (PCM) as implemented in the Gaussian 09 package [37]. In PCM, the solute is embedded into a cavity surrounded by solvent described by its dielectric constant, ε (e.g., for methanol ε = 32.6) [38]. The use of an explicit solvent has been investigated notably by Guerra *et al.*, who concluded with a better description of the electronic properties using PCM compared to the explicit solvent [39]. Hybrid models (*i.e.*, one or two molecules in the surrounding of the OH groups + PCM) were also tested for quercetin, an antioxidant, showing only slight differences in terms of BDE when compared to pure PCM calculations, while the computational time was dramatically increased [15]. All theoretical calculations, including ground state geometry optimization and frequency analysis calculations, were performed with the Gaussian 09 package [37].

Simple and multiple linear regression (SLR and MLR) analyses were used to determine regression equations, correlation coefficients *R*^2^, adjusted *R*^2^ and standard deviations (SD). PCA and HCA were employed to reduce dimensionality and investigate the subset of descriptors that could be more effective for classifying the isolated compounds according to their degree against free radicals.

The regression models and statistical analyses of the obtained results were carried out by using the DataLab package (Epina GmbH, Pressbaum, Austria).

## 3. Results and Discussion

### 3.1. Simple and Multiple Linear Regressions

The values of the calculated descriptors, as well as the logIC_50_ of the 30 Schiff bases are shown in Table 2. From the logIC_50_ (or IC_50_ value in Table 1), the Schiff bases are classified into two groups: (i) active Schiff bases with logIC_50_ < 0.3 (or IC_50_ < 2 μg/mL); and (ii) inactive Schiff bases with logIC_50_ > 0.3 (or IC_50_ >> 2 μg/mL). To determine the main descriptors that influence the antioxidant activity, first simple linear regression curves between log(IC_50_) and each descriptor were determined (figures not shown). Table 3 represents the correlation coefficients, adjusted correlation coefficients and the standard deviations obtained with each descriptor separately. The influence of each descriptor separately on the antioxidant activity mainly depends on the nature of descriptor itself (Table 3). For instance, weak correlation obtained with: (i) steric descriptors A and V with *R*^2^ < 1% and SD > 0.28; and (ii) χ, α, μ, M, IPd, IP descriptors with 4 < *R*^2^ < 14% and 0.27 < SD < 0.28. Moderate correlations are obtained with BDE, BDEd, ΔG, SD, EA and nOH descriptors with 20 < *R*^2^ < 46% and 0.21 < SD < 0.25. The importance of BDE, BDE_d_ and nOH on the antioxidant activity are consistent with those obtained in our previous study and by Amić *et al.* [13,40].

In an attempt to obtain better correlations between calculated descriptors and the observed antioxidant activity, we applied MLR analysis by combining different descriptors. The following model was obtained in PCM solvent:

log(IC_50_)_Obs._ = (−798.27 ± 609.74) − (0.81 ± 0.39) BDE + (0.04 ± 0.02) BDE_d_ − (2.73 ± 1.43) IPd − (446.44 ± 200.76) EA + (251.50 ± 165.87) χ + (3468.12 ± 2565.84) S + (168.68 ± 63.59) ω + (0.11 ± 0.07) μ + (0.0025 ± 0.0038) M − (0.95 ± 0.40) logP − (0.84 ± 0.38) *n*OH + (0.79 ± 0.35) ΔG (1)

**Table 2:** Values of the calculated descriptors and the antioxidant activity of Schiff bases (SBs). BDE, bond dissociation enthalpy; IP, ionization potential; EA, electron affinity; S, softness; A, surface area of the molecule; V, volume; M, molecular weight.

SBs	BDE	BDE_d_	IP_d_	IP	EA	χ	η	S	ω	α	μ	A	V	M	logP	*n*OH	SD	ΔG	log(1/IC_50_)	Activity
1	83.8	88.70	6.50	6.75	2.65	4.70	4.10	0.12	2.70	307.81	10.12	346.83	368.85	270	2.87	1	0.27	7.5	−0.05	Active
2	87.1	112.10	7.10	6.87	2.67	4.77	4.20	0.12	2.71	305.92	8.13	349.44	369.86	270	2.87	1	0.37	10.8	0.04	Active
3	83.2	86.10	6.40	6.62	2.54	4.58	4.07	0.12	2.58	311.99	7.65	350.17	371.21	270	2.87	1	0.32	6.8	−0.19	Active
4	77.5	76.70	6.50	6.76	2.64	4.70	4.12	0.12	2.68	314.16	9.14	356.43	379.80	286	2.58	2	0.31	1.5	−0.66	Active
5	82.2	94.10	6.40	6.50	2.52	4.51	3.97	0.13	2.56	320.20	12.12	358.17	381.33	286	2.58	2	0.25	5.7	−0.47	Active
6	78.8	68.80	6.20	6.52	2.69	4.60	3.84	0.13	2.76	315.42	11.00	359.45	382.83	286	2.58	2	0.31	2.3	−0.04	Active
7	76.1	75.90	6.20	6.52	2.55	4.54	3.97	0.13	2.59	317.53	6.18	359.09	382.23	286	2.58	2	0.25	0.2	−0.70	Active
8	86.6	107.90	7.10	6.92	2.68	4.80	4.24	0.12	2.72	312.67	8.92	361.32	383.45	286	2.58	2	0.34	10.3	−0.04	Active
9	83	83.60	6.40	6.48	2.42	4.45	4.06	0.12	2.44	326.76	8.29	367.78	392.97	302	2.3	3	0.23	6.6	−0.46	Active
10	72.3	76.60	6.20	6.51	2.57	4.54	3.94	0.13	2.62	325.00	5.73	368.00	393.67	302	2.3	3	0.27	-2.7	−0.52	Active
11	97.7	-	-	6.86	2.65	4.75	4.21	0.12	2.68	322.98	8.68	372.50	395.67	284	2.9	0	0.5	20.5	>0.30	Inactive
12	95.6	-	-	6.56	2.54	4.55	4.02	0.12	2.58	330.86	7.50	373.23	397.38	284	2.9	0	0.46	18.5	>0.30	Inactive
13	95.60	-	-	6.5	2.56	4.6	4	0.12	2.60	353.49	7.17	400.14	430.15	314	2.65	0	0.46	18.2	0.30	Inactive
14	97.7	-	-	6.86	2.65	4.75	4.21	0.12	2.69	346.83	7.67	404.68	433.54	314	2.65	0	0.5	20.4	>0.30	Inactive
15	84.1	86.30	6.40	6.48	2.53	4.51	3.95	0.13	2.57	338.44	8.42	381.97	408.56	314	2.61	1	0.28	7.7	−0.30	Active
16	79.2	91.80	6.10	6.49	2.67	4.58	3.82	0.13	2.75	331.70	10.35	382.84	408.79	314	2.61	1	0.31	0	0.00	Active
17	86	100.60	6.30	6.47	2.55	4.51	3.92	0.13	2.60	336.25	6.16	381.75	408.07	300	2.61	1	0.33	9.3	−0.10	Active
18	82.2	86.80	6.40	6.74	2.63	4.68	4.10	0.12	2.67	341.29	6.44	373.11	399.32	349	3.66	1	0.26	6	−0.66	Active
19	99	-	-	7.04	2.85	4.95	4.19	0.12	2.92	351.56	9.76	380.01	407.79	367	4.46	0	0.53	21	>0.30	Inactive
20	97.4	-	-	6.93	2.66	4.79	4.26	0.12	2.70	297.87	10.32	346.15	367.00	272	3.29	0	0.49	20.4	>0.30	Inactive
21	97.7	-	-	6.95	2.75	4.85	4.20	0.12	2.80	321.19	10.50	358.15	380.50	289	3.67	0	0.49	20.7	>0.30	Inactive
22	97.80	-	-	-	-	-	-	-	-	-	-	-	-	-	-	-	-	-	-	Inactive
23	98.6	-	-	7.10	3.07	5.08	4.03	0.12	3.21	353.21	12.32	424.58	398.21	312	2.86	0	0.45	21.3	>0.30	Inactive
24	99.6	-	-	7.25	3.83	5.54	3.41	0.15	4.50	350.66	14.25	368.39	393.25	299	3.11	0	0.5	21.2	>0.30	Inactive
25	97.5	-	-	6.94	2.67	4.81	4.28	0.12	2.70	299.19	9.46	338.81	358.66	254	3.15	0	0.51	20.3	>0.30	Inactive
26	98.4	-	-	7.15	2.86	5.00	4.29	0.12	2.92	291.63	11.56	334.13	352.57	255	2.7	0	0.57	20.8	>0.30	Inactive
27	98.5	-	-	7.11	2.80	4.96	4.31	0.12	2.85	289.70	12.62	333.11	351.97	255	2.77	0	0.51	21.2	>0.30	Inactive
28	99.4	-	-	7.26	2.97	5.12	4.29	0.12	3.05	289.74	11.55	333.31	352.15	255	2.77	0	0.54	21.7	>0.30	Inactive
29	94.9	-	-	6.67	2.64	4.65	4.03	0.12	2.68	275.78	10.26	319.70	333.11	244	1.79	0	0.45	18	>0.30	Inactive
30	95.1	-	-	6.72	2.73	4.73	3.99	0.13	2.80	297.67	10.09	328.48	346.48	260	2.13	0	0.44	18.2	>0.30	Inactive

**Table 3:** Correlation coefficients, adjusted correlation coefficients and standard deviations correspond to the simple linear regressions between logIC_50_ and calculated descriptors.

Descriptors	R^2^ (%)	R^2^ Adjusted	SD
BDE	31.18	25.45	0.2372
BDE_d_	28.26	22.28	0.2422
IP_d_	11.47	4%	0.2691
IP	3.88	−4%	0.2804
EA	20.57	13.95	0.2549
χ	9.58	2.05	0.2719
η	0.1	−8.23	0.2858
S	0.28	−8.03	0.2856
ω	21.98	15.48	0.2526
α	8.43	0.7	0.2738
μ	13.07	5.82	0.2666
A	0.34	−7.96	0.2855
V	0.91	−7.34	0.2847
M	14.42	7.29	0.2645
logP	0.33	−7.98	0.2855
*n*OH	20.46	14.03	0.2548
SD	46.35	41.88	0.2095
ΔG	21.78	15.26	0.2529

**Figure 1:**
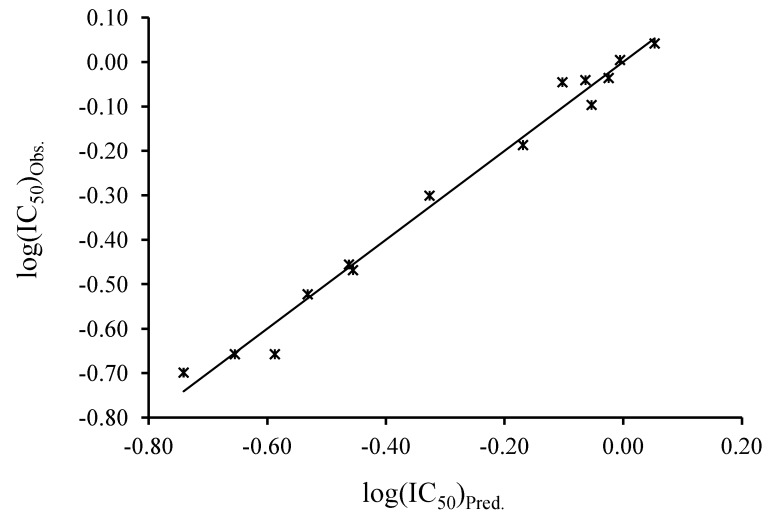
Multiple linear regression curve between the log(IC_50_)_Obs._ and log(IC_50_)_Pred._ of the active Schiff bases.

The above model (Equation 1) was obtained between log(IC_50_)_Obs._ and the main descriptors of the active Schiff bases (Figure 1). The predicted log(IC_50_)_Pred._ and residuals to experimental log(IC_50_)_Obs._ for the 14 active Schiff bases are shown in Table 4. The correlation between the descriptors and the log(IC_50_)_Obs._ is relatively good, with *R*^2^ = 98.57%, *R*^2^ adjusted = 81.46% and SD = 0.12. The standard deviation of the model of Equation 1 is better than the ones obtained with separated descriptors. In an attempt to reduce the number of descriptors, the Equations 1 and 2 models were obtained with three and four descriptors, respectively.

log(IC_50_)_Obs._ = (242.10 ± 78.30) + (0.05 ± 0.01) BDE + (29.37 ± 9.77) η + (989.33 ± 312.49) S + (0.01 ± 0.00) α (2)

log(IC_50_)_Obs._ = −(8.36 ± 1.64) + (0.05 ± 0.01) BDE + (2 ± 0.53) μ − (0.40 ± 0.15) logP (3)

The predicted log(IC_50_)_Pred._ and residuals to experimental log(IC_50_)_Obs._ for the 14 active Schiff bases obtained with the Equations 2 and 3 models are shown in Table 4. The correlation coefficients obtained with Equations 2 and 3 regression equations are 84.4 and 74%, respectively. The corresponding standard deviations are 0.13 and 0.16 for Equations 2 and 3, respectively. The model of Equation 2 showed the importance of BDE, η, S and polarizability α descriptors, while the model of the Equation 3 showed the importance of BDE, μ and logP on the antioxidant activity. The antioxidant activity is important for Schiff bases with low BDE values. For instance, the low antioxidant activity of **2** is in agreement with the relatively high BDE of the ortho-2-OH group (87.1 kcal/moL) compared to those of the meta-3-OH and para-4-OH groups (83.8 and 83.1 kcal/moL, respectively). LogP is a measure of the hydrophobicity. As can be observed in Table 1, the most active Schiff bases have low logP values. Indeed, the Schiff bases containing more OH groups are more soluble in water, and they are hydrophilic. For instance, the Schiff bases, **1**–**3**, with one OH group (logP = 2.87), are less active than the Schiff bases, **4**–**8**, with two OH groups (logP = 2.58), which are less active than the Schiff bases, **9**, **10**, with three OH groups (logP = 2.3). Somehow, these results are in good agreement with previous studies, which showed the importance of logP as a descriptor to classify compounds into high and low activities [41,42].

**Table 4:** Experimental and predicted log(IC_50_) for the active Schiff bases (SBs).

SBs	log(IC_50_)_Obs._	Equation 1			Equation 2			Equation 3	
		log(IC_50_)_Pred._	Residual		log(IC_50_)_Pred._	Residual		log(IC_50_)_Pred._	Residual
**1**	−0.05	−0.10	−0.06		−0.22	−0.17		−0.15	−0.10
**2**	0.04	0.05	0.01		0.02	−0.02		0.03	−0.02
**3**	−0.19	−0.17	0.02		−0.28	−0.09		−0.42	−0.23
**4**	−0.66	−0.59	0.07		−0.57	0.09		−0.36	0.30
**5**	−0.47	−0.46	0.01		−0.29	0.18		−0.38	0.09
**6**	−0.04	−0.02	0.01		0.02	0.06		−0.14	−0.10
**7**	−0.70	−0.74	−0.04		−0.54	0.16		−0.60	0.10
**8**	−0.04	−0.06	−0.02		−0.01	0.03		0.14	0.18
**9**	−0.46	−0.46	−0.01		−0.43	0.03		−0.47	−0.01
**10**	−0.52	−0.53	−0.01		−0.72	−0.20		−0.62	−0.10
**15**	−0.30	−0.33	−0.03		−0.33	−0.03		−0.28	0.02
**16**	0.00	−0.01	−0.01		−0.04	−0.04		−0.16	−0.16
**17**	−0.10	−0.05	0.04		−0.13	−0.04		−0.14	−0.05
**18**	−0.66	−0.65	0.00		−0.62	0.03		−0.59	0.07

### 3.2. Principal Component Analysis (PCA)

The PCA method allows the reduction of the number of variables used in the statistical analysis and creation of a new set of variables (PCs) expressed as a linear combination of the original data set [43]. The first new variable (PC1) contains the largest variance; the second contains the second largest variance, and so on. Before applying the PCA method, each variable was standardized, so that they could be compared to each other on the same scale. After several attempts to obtain a good classification of the synthesized Schiff bases, the best separation was achieved with three variables, BDE, μ and logP. The first two components of PCA describe 97.75% of the overall variance of the data set (Table 5), in which the PC1 describes 77% of that variance. The loading vectors for PC1 and PC2 are displayed in Table 6.

**Table 5:** Variances (eigenvalues) obtained for the first two principal components.

Component	Eigenvalue	Variance (%)	Cumulated Variance (%)
PC1	2.306	76.88	76.88
PC2	0.6261	20.87	97.75

**Figure 2:**
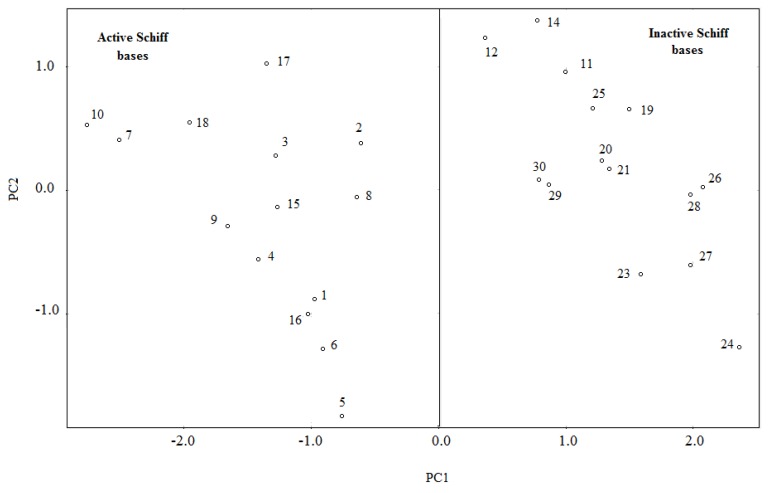
Plot of the first two principal components (PC) score vectors for Schiff bases.

The plot of the score vectors of the two principal components (PC1 × PC2) is shown in Figure 2. From Figure 2, the Schiff bases are separated into two groups: the active (**1**–**10**, **15**–**18**) and the inactive Schiff bases (**11**–**14**, **19**–**30**). From Table 6, the principal component, PC1, can be expressed through the following equation:

PC1 = 0.63IP + 0.47 BDE + 0.46 μ + 0.62 logP(4)

From the above equation (Equation 4), for a Schiff base to be active, it has to have positive values of BDE, μ and logP.

**Table 6:** Loading vectors for the first two principal components.

Variable	PC1	PC2
BDE	0.6276	0.3014
μ	0.4724	−0.8803
logP	0.6189	0.3663

### 3.3. Hierarchical Cluster Analysis (HCA)

**Figure 3:**
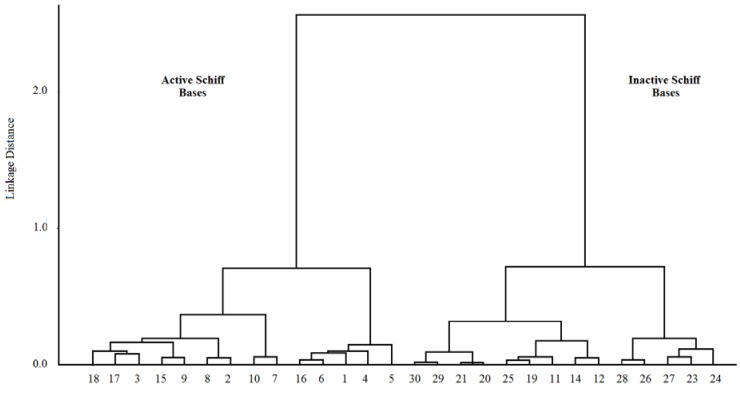
Dendrogram obtained with hierarchical cluster analysis (HCA) for Schiff bases.

For preliminary data analysis, the HCA method is a powerful tool for examining data sets for expected or unexpected clusters, including the presence of outliers. It examines the distances between the samples in a data set and represents them in a dendrogram [44]. HCA gives similar information as PCA results. In the HCA analysis, each point (Schiff base) forms an only cluster, and then, the similarity matrix is analyzed. The most similar points are assembled, forming one cluster, and the process is repeated, until all the points belong to only one group [44]. The results obtained with the HCA analysis in the dendrogram are displayed in Figure 3; the vertical lines represent the compounds, and the horizontal lines represent the distances between a pair of compounds, a compound and a group of compounds and between groups of compounds. The Schiff bases are subdivided into two groups according to the distance: (i) active and (ii) inactive Schiff bases. These two groups are similar to those obtained with PCA methods.

## 4. Conclusions

The combination of statistical methods (SLR, MLR, PCA and HCA) and quantum chemical calculations allows a better description of the structure antioxidant activity relationships of a series of 30 Schiff bases. Based on the descriptors’ contributions, the Schiff bases are subdivided into active and inactive subclasses.
